# Continuous Wavelet Transform-Based Method for High-Sensitivity Detection of Image Signals of Fluorescence Lateral Flow Assay

**DOI:** 10.3390/s25133846

**Published:** 2025-06-20

**Authors:** Tao Zhang, Xiaosong Wu, Qian Wang, Long Zhang, Zhigang Li, Yangyang Peng, Qian Bian, Hui Shi, Yong Liu, Shu Wang

**Affiliations:** 1 School of Biomedical Engineering, Anhui Medical University, Hefei 230032, China; zhangtao1230906@163.com (T.Z.);; 2 Hefei Institute of Physical Science, Chinese Academy of Sciences, Hefei 230036, China; wxs@aiofm.ac.cn (X.W.);

**Keywords:** fluorescence lateral flow assays, trace detection, filters, continuous wavelet transform, weak fluorescence signals

## Abstract

Fluorescence lateral flow assays (FLFA) based on quantum dot probes have attracted significant attention in recent years due to their high sensitivity and quantitative detection capabilities. FLFA requires the use of a straightforward fluorescence reader for quantitative detection. Most fluorescence readers employ narrowband filters for auxiliary imaging, which facilitates the acquisition of high-contrast signals. However, during trace detection, the weak signal from FLFA can be easily lost due to optical flux loss associated with narrowband filters, thereby indirectly diminishing detection sensitivity. To address this issue, we developed a fluorescence signal reader that employs CMOS imaging without optical filters and proposed a highly sensitive signal detection algorithm based on continuous wavelet transform (CWT) to identify weak fluorescence signals with low contrast. Experimental results demonstrate that the method achieves a fluorescence detection sensitivity for quantum dots of 10^−10^ mol/L, with a relative standard deviation (RSD) of < 1.45%. The designed filter-free detection system and CWT analysis algorithm were applied to various FLFA systems (including the sandwich method and the competition method), with the correlation coefficient (R^2^) between all detection results and sample concentration exceeding 0.997. The findings of this study offer a highly sensitive signal detection method for the precise quantification of FLFA.

## 1. Introduction

In recent years, the COVID-19 pandemic, along with influenza and other infectious diseases, has resulted in significant human and economic losses on a global scale [1,2,3]. In epidemic-prevention efforts across various regions, low-cost lateral flow assay test strips are widely utilized for rapid onsite screening [4,5]; however, insufficient sensitivity limits their further application [6]. To address this limitation, some researchers have proposed using composite quantum dot microspheres with high-intensity fluorescence signals as detection labels, rather than colloidal gold, resulting in significant breakthroughs [7]. Lateral flow assays utilizing fluorescence quantum dot tags must be combined with fluorescence reading instruments for high-sensitivity quantitative detection. Existing fluorescence reading instruments primarily rely on imaging measurement methods [8], which employ CCD/CMOS cameras to collect fluorescence images and subsequently utilize image processing algorithms to identify and analyze the T-line area [9]. Consequently, the quality of signal acquisition and the effectiveness of signal analysis in the fluorescence reader will directly impact the detection results of fluorescence lateral flow assays (FLFA). To further enhance detection accuracy and mitigate noise, researchers frequently employ a variety of denoising algorithms. Among these methods, the wavelet transform, which offers joint time–frequency localization, has been widely employed for image denoising and weak-signal extraction [10].

When developing FLFA signal readers, it is a standard practice to employ narrowband filters that correspond to the emission wavelength of quantum dot tags for auxiliary imaging. The primary types of filters are absorption-based, interferometric, and holographic filters [11]. Due to variations in working principles, materials, and structures, transmittance loss differs among filters: (1) The principle of the absorptive narrowband filter involves using materials with specific absorption characteristics to absorb light outside a particular wavelength, resulting in transmittance loss between 30% and 50% [12]. (2) The principle of the interferometric narrowband filter involves utilizing the interference phenomenon of light between multilayer films to achieve narrowband filtering. Its transmittance loss ranges from 5% to 20%, and its performance requires a highly precise film preparation process [13]. (3) Holographic narrowband filters employ periodic holographic gratings to achieve selective transmission of light waves at specific wavelengths, with transmittance loss ranging from 10% to 30%. The structure of holographic gratings is susceptible to ambient light and temperature variations, which can result in decreased transmittance [14]. Although high-purity optical materials, optimized film design, preparation processes, and anti-reflective treatments on the surface of the filter can enhance the transmittance of narrowband filters, they cannot completely eliminate transmittance loss when used to collect optical signals within the specified band [15,16]. Therefore, when employing FLFA for high-sensitivity detection of trace targets, the phenomena of signal attenuation or complete loss caused by narrowband filtering are inevitable; consequently, it becomes challenging to identify and locate the contrast signal of the T-line by directly collecting fluorescence images of the test strip without utilizing a filter. As a result, filter-assisted imaging methods are unsuitable for high-sensitivity detection of trace targets; thus, it is crucial to achieve the identification and analysis of low-contrast weak signals in the original images.

Numerous studies have investigated fluorescence dipstick signal analysis algorithms. In 2019, Guo et al. developed a region-growing algorithm combined with fast peak detection (RGFPD) [17] for adaptive segmentation of test strip images. In 2019, Jiang et al. developed an image entropy-based Otsu segmentation algorithm that demonstrated good adaptability and resistance to variations in gray values and offset effects [18]. Zeng et al. proposed employing the gradient projection algorithm to denoise and deblur immune tomography detection strip images to enhance the accuracy of signal reading [19]. Although these lateral flow assays’ image signal detection algorithms have demonstrated good performance in their respective studies, they primarily target high-concentration samples with significant contrast, and only a limited number of low-concentration samples are involved. Therefore, analyzing weak fluorescence signals from test strips remains a challenging problem, which limits the applicability of FLFA in various scenarios. To achieve high-sensitivity detection and accurate quantification of low-contrast weak fluorescence signals, we developed a filterless dipstick fluorescence signal reader and proposed a high-sensitivity detection algorithm based on continuous wavelet transform (CWT). The algorithm employs the CWT function to enhance signal contrast in the directional projection curve of the dipstick. Accurate identification of weak signal regions is achieved by combining first derivative and zero-crossing point detection. The performance of the developed method was simultaneously validated in various flux modes (single/double channel) and assay types (sandwich/competition) in FLFA.

## 2. Materials and Methods

The materials and reagents used in this work are described in Supporting Information S1.

### 2.1. FLFA Signal Detection System

**Figure 1:**
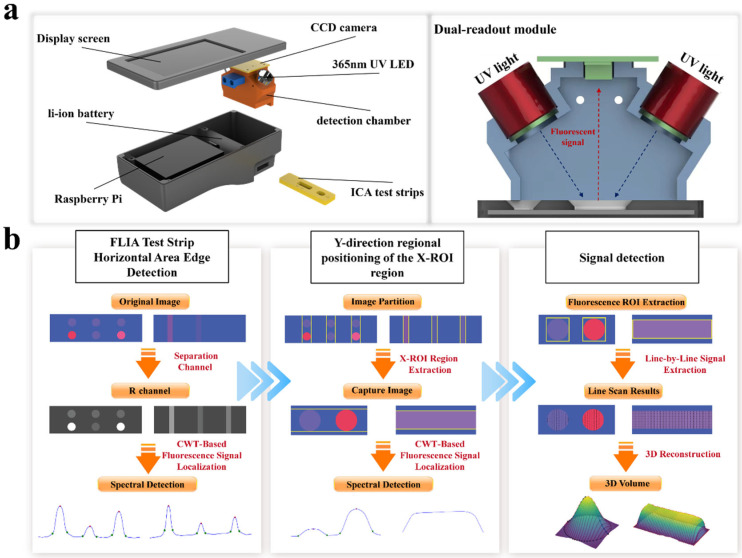
FLFA detection system. (**a**) Schematic diagram of the overall structure of the fluorescence quantitative detector and the detection optical path design. (**b**) Flowchart of the high-sensitivity detection method of the FLFA image signal based on CWT.

Figure 1a displays the main structure of the FLFA comprehensive reading instrument. This device utilizes a Raspberry Pi development board (Raspberry Pi 5 BCM2712 quad-core 64-bit Arm Cortex-A76 CPU, SONY UK Factory., Ltd., UK) and is equipped with portable FLFA detection software (FLFA reads system V1.0) designed for the Raspberry Pi OS, integrated with a test strip detection chamber, and powered by a lithium battery. The structure of the fluorescent test strip detection chamber is shown in Figure 1b, with the chamber component fabricated using 3D printing technology.

In the optical detection system, excitation light is supplied by a SVC ultraviolet LED (Tecsun Electronics Co., Ltd., Seoul, Republic of Korea, emission wavelength: 365 nm, power: 3 watts, incident angle: 45 degrees) to excite the quantum dot (QD, the spectral characteristics are shown in Supplementary Material S13) labels on the FLFA strip, facilitating the acquisition of fluorescent signals. The collimator ensures that excitation light from a single LED passes through a narrow-band filter to reach the detection area while effectively blocking scattered light from other sources. Additionally, the device includes a Sony ICX285 CCD camera (SONY., Ltd., Tokyo, Japan) with an autofocus lens, an exposure time set to 1/100 s, and which acquires images without a filter (See Supplementary Material S12 for details).

In the actual detection process, the user first opens the software and selects the detection mode. Subsequently, the test strip is inserted into the detection chamber, where a built-in contact photoelectric switch automatically activates the LED photodiode. The generated excitation light illuminates the test strip through a 365 nm narrow-band filter (BP365-40k, Tangxue PHOTOelectric Technology Co., Ltd., Shijiazhuang, China, the spectral characteristics are shown in Supplementary Material S13), which causes the nano labels to emit red, fluorescent signals. Finally, the user presses the capture button in the software to activate the CCD camera and capture the image of the fluorescent test strip; an image detection algorithm is then employed to automate the analysis of the fluorescent signal values.

A high-sensitivity fluorescence dipstick signal detection algorithm based on the continuous wavelet transform (CWT) is introduced. In this process, both vertical and horizontal projections of the fluorescence signal are utilized to adequately capture the signal distribution throughout the detection area, as shown in Figure 1b. The Mexican Hat wavelet function is applied in the convolution processing of the projection curve to enhance the signal-to-noise ratio and accurately extract the fluorescence signal region. The combination of vertical and horizontal projections ensures a comprehensive analysis of the signal profile, allowing for the precise identification of fluorescence regions in both dimensions.

Subsequently, the first derivative analysis and trough detection are combined to ensure the accuracy of identifying the signal peak boundary along the projection direction. For each detected fluorescence signal region, the algorithm progressively scans to extract the peak area, generating a three-dimensional (3D) model of the fluorescence signal through integral reconstruction from the two projections. When handling images with a low signal-to-noise ratio, bright backgrounds, or increased noise, the proposed method effectively preserves signal features, filters out noise, improves peak location accuracy, and enables highly sensitive fluorescence signal detection.

### 2.2. High-Sensitivity Detection Algorithm for Fluorescent Signals Based on CWT

**Figure 2:**
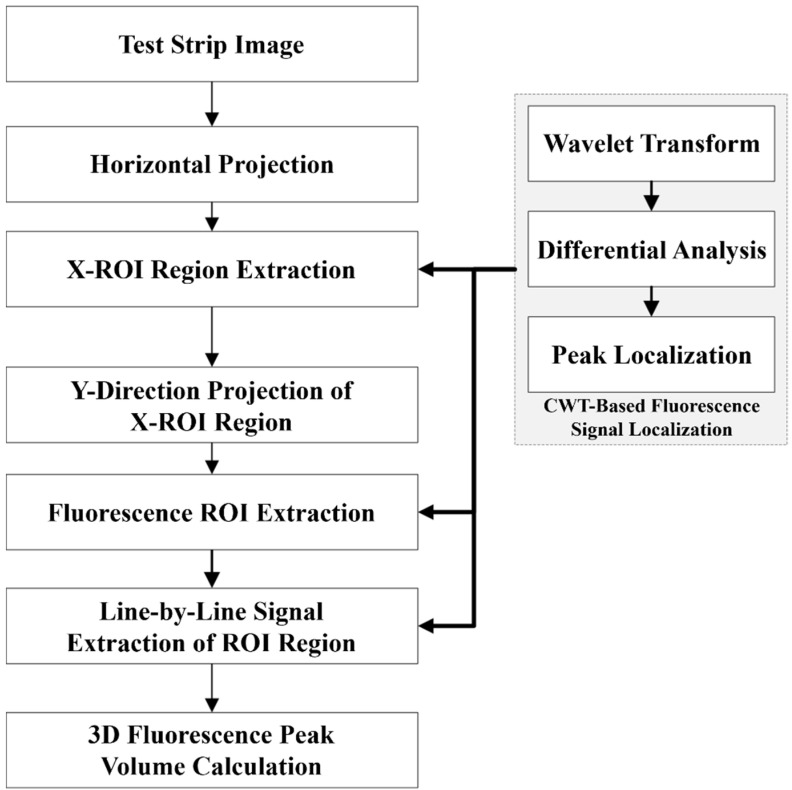
Flowchart of high-sensitivity detection algorithm of fluorescence signal based on CWT.

This paper introduces a high-sensitivity detection algorithm utilizing CWT that enhances the ability to detect and quantify weak fluorescence signals by effectively filtering noise and accurately identifying signal regions. The basic workflow of the algorithm is depicted in Figure 2. Initially, the FLFA strip image is processed through two stages: (1) transverse projection positioning and (2) longitudinal projection positioning, both based on CWT. Subsequently, the fluorescence intensity of each signal region is determined using the CWT-based fluorescence signal analysis method.

### 2.3. Basic Principle of CWT

The fundamental principle of CWT is to analyze the local features of signals across different time scales using wavelet functions, enabling simultaneous analysis in both the time and frequency domains [20]. This time-frequency localization allows CWT to effectively capture instantaneous changes in the signal and distinguish subtle differences between signal and noise, particularly in low signal-to-noise ratio (SNR) environments. Consequently, applying CWT to fluorescence curve analysis in low SNR conditions helps preserve the primary characteristics and morphology of the signal while effectively filtering out high-frequency noise and mitigating other artifacts. For a given input signal *x*(*t*), the CWT is defined in Equation (1):

(1) $$W\left(s,\tau \right)=\frac{1}{\sqrt{\left|s\right|}}\int_{-\infty }^{+\infty }x\left(t\right)\psi^{*}(\frac{t-\tau }{s})dt$$

where *t* represents the number of rows of the signal, *s* is the wavelet scale (inversely related to frequency), and *τ* specifies the temporal translation of the wavelet. The function *ψ*(*t*) denotes the wavelet function, *ψ^∗^(t*) represents the complex conjugate of *ψ*(*t*), and *x*(*t*) is the input signal in the time domain.

### 2.4. FLFA Image Directional Projection Curve

The directional projection curve of a fluorescence image is a one-dimensional signal representation method created by summing the gray values of pixels along a specified direction within the image. This technique simplifies the image information and accentuates the fluorescence intensity distribution. By compressing the data from a two-dimensional image into a one-dimensional signal, the projection process facilitates a more intuitive and convenient analysis of both the overall trend and local characteristics of the fluorescence signal. The projection curve is given in Equation (2):

(2) $$Y_{j}=\sum_{i=1}^{h}y_{ij},\left(j=1,2,\cdots ,w\right)$$

where h denotes the number of rows projected by the fluorescence image, w represents the width of the image along the measurement direction, and *Y_j_* indicates the signal intensity calculated by summing the gray values of pixels in the *j*-th column from row 1 to row h on the fluorescence image.

### 2.5. Peaks Location Based on CWT

Prior to waveform projection analysis, the CWT and curve crest positioning are applied to process the original waveform, significantly improving the signal-to-noise ratio (SNR) and thereby enhancing the accuracy of subsequent peak detection. In this paper, the Mexican Hat wavelet function is employed. Due to the absence of lobes with values greater than 0 on both sides of the Mexican Hat wavelet function, it does not produce direct positive interference peaks. Furthermore, by systematically comparing various wavelet basis functions and scale ranges, we found that the Mexican Hat wavelet applied at scales 30–37 (decomposition level 128) produced the greatest noise reduction in FLFA signals (Supplementary Materials S8 and S11). The projection curve is convolved with this wavelet function. The formula for the wavelet function is provided in Equation (3):

(3) $$\psi \left(t\right)=(1-\frac{t^{2}}{\sigma^{2}})exp(-\frac{t^{2}}{2\sigma^{2}})$$

where *σ* represents the scale factor controlling the width of the wavelet, *t* denotes the time parameter or the local position parameter of the signal, and *ψ*(*t*) refers to the Mexican Hat wavelet function.

**Figure 3:**
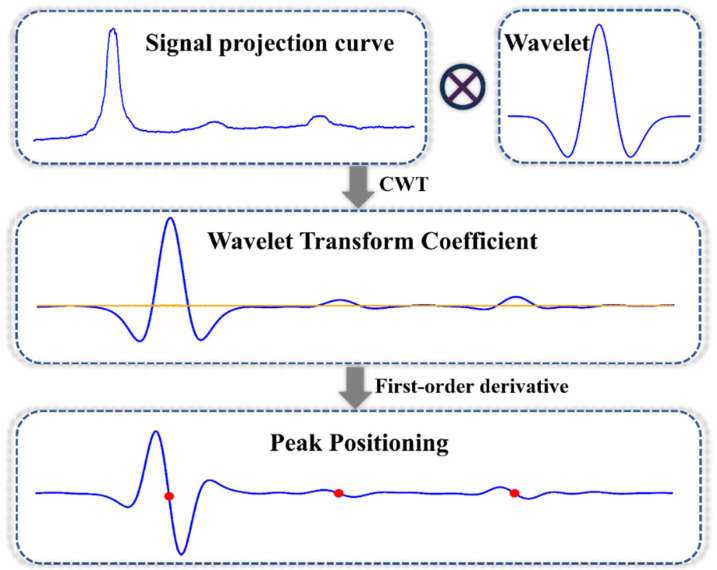
Schematic diagram of fast localization algorithm based on CWT and local peak signal (In the figure, the orange line of the wavelet coefficient is the zero value line, and the red dot of the first derivative is the zero crossing point).

Figure 3 illustrates the principle of the peak-seeking algorithm. The first derivative of the signal curve, *W*(*j*), after wavelet transformation is calculated. The corresponding principle is presented in Equation (4):

(4) $$W_{j}^{\prime }=\frac{W\left(j+n\right)-W(j-n)}{2n+1},\left(j\in \left[n,w-n\right]\right)$$

where *W_j_*′ denotes the first derivative of *W_j_*, *n* represents the half-width of the derivative interval, and for each point *j* on the transverse axis of the derivative curve *W_j_′*, the zero-crossing points of the peaks and troughs are identified according to Equation (4). Using the wavelet transform in combination with differential extreme-value detection, the position of the positive peak is determined by applying a derivative sign-change constraint.

Once a peak is detected, the nearest troughs are identified by tracing back to the left and right; these troughs are subsequently utilized as the left and right boundaries of the peak. In practical applications, noise within the signal and interference from extraneous peaks may result in erroneous trough detection. Consequently, a threshold criterion is established to delineate the left and right boundaries. For instance, a boundary is deemed valid only if the difference in distance between the left and right troughs surpasses a specified threshold. Finally, the baseline was computed as the arithmetic mean of the left- and right-boundary values. This principle is illustrated in Equation (5):

(5) $$\left\{\begin{array}{c} baseval=\frac{Y\left(j_{left}\right)+Y(j_{right})}{2}\\ j_{right}-j_{left}>\mu \end{array}\right.$$

where *baseval* denotes the baseline value of the peak, and *Y(j_left_)* and *Y(j_right_)* correspond to the amplitudes of the troughs on the left and right sides, respectively. Meanwhile, *j_left_* and *j_right_* indicate the positions of the left and right trough points, and *μ* represents the defined threshold. In our detection algorithm, the discrimination threshold *μ* for identifying effective peaks is set at 32 (for details, see Supplementary Information S10).

### 2.6. Fluorescence Region Extraction

The projection method outlined in Section 2.4 was applied to generate the projection curve of the FLFA strip image along the X direction, and the ROI region was identified and extracted using the approach described in Section 2.5. Subsequently, the ROI region is projected in the Y-direction. If no peak is detected in the projection curve, the Y-direction projection region is assumed to contain only strip fluorescence, and the projection region is classified as the fluorescent ROI region. If a peak is present, the projection region should be identified and segmented based on the method in Section 2.5, yielding the ROI image of the fluorescence region.

### 2.7. Fluorescence Signal Quantification Based on CWT Peak Integral Volume

**Figure 4:**
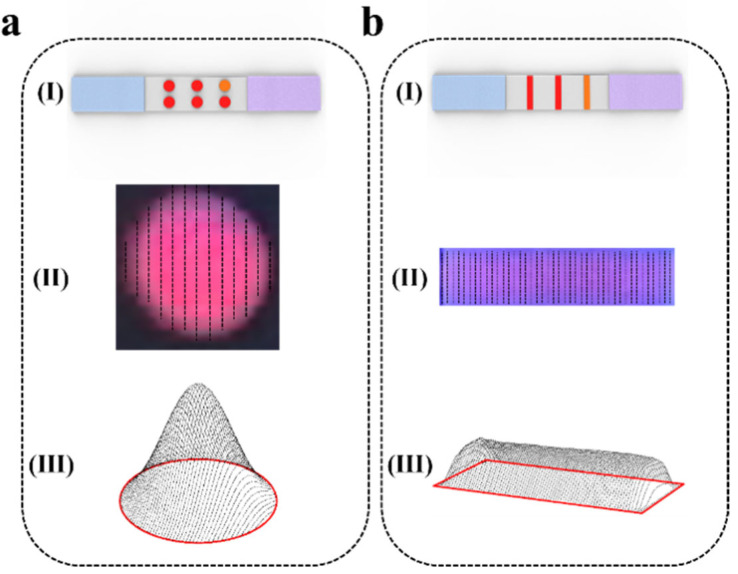
Schematic representation of signal quantification in the fluorescent ROI region. (**a**) Dot matrix strip scanning reconstruction. (**b**) Strip scanning reconstruction. (**I**) Schematic diagram of strip scanning. (**II**) progressive scanning of ROI region (**III**) integral reconstruction of ROI region.

After obtaining the ROI image of each fluorescence signal on the dot matrix or strip test strip (Figure 4(a(I), b(I))), we scanned it step by step and obtained the peak area of the fluorescence signal in each scan line using the wavelet-transform-based peak location method described in Section 2.4 (Figure 4(a(II), b(II))). After the step-by-step scanning was completed, all the extracted peak curves were integrated and reconstructed to obtain a three-dimensional model of the fluorescence signal (Figure 4(a(III), b(III))). The intensity of the fluorescence signal in the ROI image is represented as the three-dimensional volume of the fluorescence signal.

(6) $$avePix=\frac{1}{\sum_{y=down}^{top}\left({dp}_{r}\left(y\right)-{dp}_{l}(y)\right)}\sum_{y=down}^{top}\left(\sum_{x={dp}_{l}(y)}^{{dp}_{r}(y)}\left(f\left(x,y\right)-{baseval}_{y}\right)\right)$$

where *dp_l_*(*y*) and *dp_r_*(*y*) represent the left and right trough points for row *y* after progressive scanning, *top* and *down* refer to the left and right boundaries obtained by projecting the one-dimensional waveform in the Y-direction of the X-ROI region, representing the maximum longitudinal range of the fluorescence region, *baseval_y_* is the baseline value of the y-row scan in the fluorescence ROI region, as defined in Equation (5), *f*(*x,y*) denotes the value at row *y*, column *x* of the one-dimensional waveform progressively scanned within the ROI region of the original image, and, finally, *avePix* represents the average fluorescence gray value.

## 3. Results and Discussion

### 3.1. Comparison Test With or Without Filters

**Figure 5:**
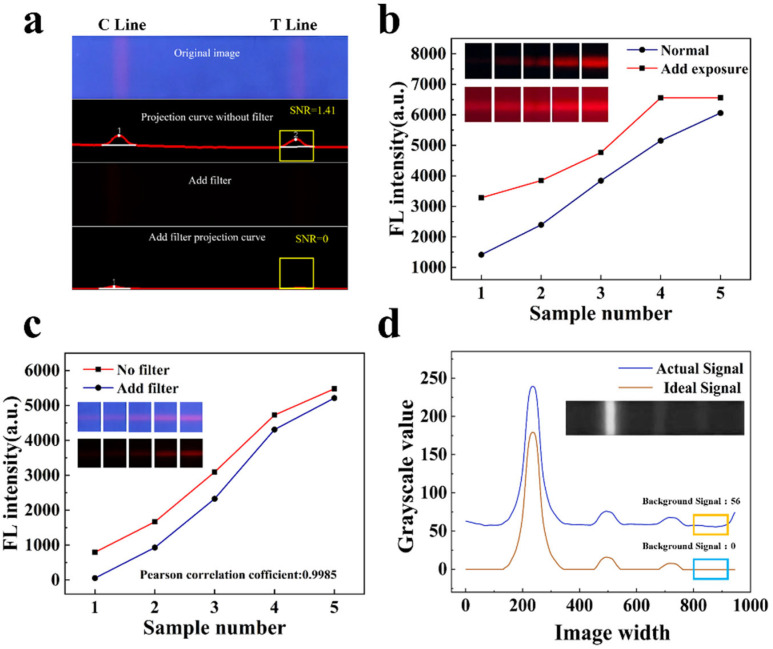
(**a**) The effect of adding a filter on the detection of weak fluorescence dipsticks (In the figure, point 1 is the C-line and point 2 is the T-line). (**b**) The effect of increased exposure on the detection results. (**c**) Comparison of detection results between no filter and added filter. (**d**) The influence of stray light on the detection signal in the actual detection.

To verify the advantages of our work in scenarios without a filter to suppress stray light, we utilized the same light source, camera, and exposure to design fluorescence signal detection for carbendazim samples at a concentration of 0.06 mg/kg (the clinically required detection limit), both without and with filters [21]. As illustrated in Figure 5a, the fluorescence signal in the target area is detectable in the image acquired without a filter, while the fluorescence signal in the target area is barely perceptible in the image obtained with a filter, with no detectable fluorescence signal in the projection curve. Adjusting only the camera exposure to enhance the collected fluorescence signal may lead to overexposure, resulting in the loss of the original gradient characteristics of the collected FLFA image, as shown in Figure 5b. Therefore, the weak fluorescence signal can be more effectively detected without the use of a filter. (The specific fluorescence values lost due to the use of filters are presented in Table S1.)

Secondly, to assess the influence of background interference caused by our detection algorithm in the absence of a filter on detection accuracy, we prepared five positive sample solutions, utilizing carbendazim antibodies modified by silicon core double-layer quantum dot (SDQD) composite nanomaterials as fluorescent probes (excitation wavelength: 365 nm). Each sample was tested both with and without a filter, and the fluorescence intensity values obtained from both methods were recorded. As presented in Figure 5c, the fluorescence signal detection algorithm we designed exhibits the same detection trend of fluorescence intensity both with and without the filter. The Pearson correlation coefficient was calculated to be 0.9985 (with values ranging from 0 to 1, where values closer to 1 indicate a higher correlation), indicating that background interference in the absence of the filter does not affect the detection results.

Fluorescence is excited in the UV region and detected in the red region, as illustrated in Figure 5d. The blue curve represents the influence of stray light on the detection signal during actual detection, while the red curve represents the fluorescence signal curve in an ideal state. It is evident that stray light causes the background signal of the fluorescent test strip to be elevated, resulting in generated noise. Consequently, the algorithm presented in this paper is required to eliminate background noise and enhance the signal-to-noise ratio, ensuring that the actual detected signal approximates the ideal signal more closely.

### 3.2. Repeatability Analysis of Fluorescence Signal Detection

**Figure 6:**
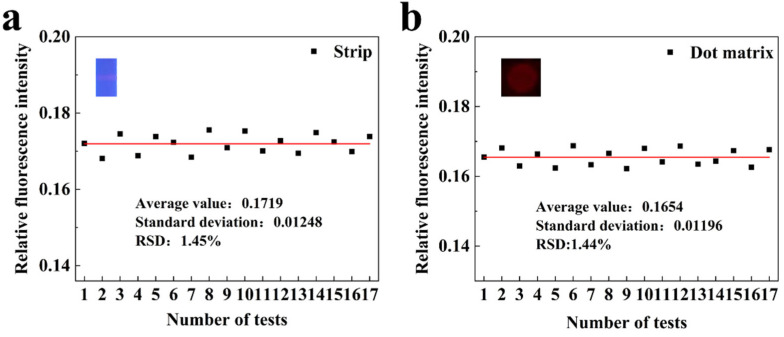
Reproducibility test of fluorescence intensity detection. (**a**) Schematic diagram of the reproducibility test for fluorescence intensity of strip test strips. (**b**) Schematic diagram of the reproducibility test for fluorescence intensity of dot matrix test strips.

To evaluate the repeatability of fluorescence intensity detection using the fluorescence quantitative reader, a monkeypox antibody modified with silicon core double-layer quantum dots (SDQD) was utilized as a fluorescent probe, with an excitation wavelength of 365 nm (for details, see Supplementary Information S2). A 0.005 ng/mL monkeypox sample solution was prepared as a target for low concentration detection and subsequently applied to two types of test strips: standard strips and dot matrices. The strips were then inserted into the instrument for seventeen repeated measurements for each sample, which recorded the corresponding relative fluorescence intensity values. The fluorescence quantitative reader conducted seventeen repeated measurements for each sample, recording the corresponding relative fluorescence intensity values. As illustrated in Figure 6a,b, the seventeen repeated measurements of the sample yielded relative fluorescence intensities for both the strip and dot matrixes test strips, with the average fluorescence value for the strip being 0.1719, a standard deviation of 0.01248, and a relative standard deviation (RSD) of 1.45%. The average fluorescence value for the dot matrices was 0.1654, accompanied by a standard deviation of 0.01196 and an RSD of 1.44%. These results indicate that the fluorescence quantitative reader demonstrates good detection repeatability. Meanwhile, the excellent detection sensitivity and accuracy of the optical system used in this study were verified by the fluorescent dye method (Figures S2 and S3).

### 3.3. Comparison Test of Peak Location Algorithms

There are many methods for peak detection, such as threshold methods, curve-fitting, dynamic programming, and Gaussian model peak localization methods [22,23,24]. In order to compare the detection accuracy and robustness of the proposed algorithm with other classical peak detection algorithms for different concentrations of FLFA signals, the carbendazim antibody modified by SDQD quantum dot material was used as a fluorescent probe (excitation wavelength 365 nm) for detection, and 50 negative sample solutions were prepared and mixed with a proper amount of fluorescent probe to drop onto the strip. The instrument was inserted for signal acquisition. The collected signals were detected using the four classical peak finding algorithms introduced above and the peak location algorithm proposed in this study. Five repeated tests were performed for each sample to record whether the location of the peak point was consistent with the actual peak position. The comparison results are shown in Table 1, from which it can be seen that the four classical detection methods perform similarly in terms of accuracy, with the proposed method in this study achieving 95% accuracy in weak signals and 98% accuracy in noise. It shows that the algorithm in this study has better accuracy and robustness in signal detection (for detailed peak discrimination, see Supplementary Information S9; see S15 for noise quantization).

### 3.4. Practical Application of the Filterless Detection System and CWT Analysis Algorithm

#### 3.4.1. Quantitative Analysis for Single-Channel FLFA

**Figure 7:**
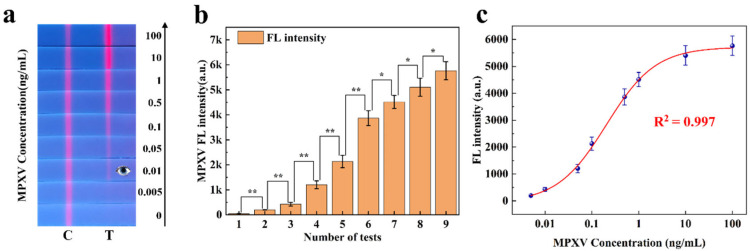
(**a**) Monkeypox detection strips with different fluorescence intensities. (**b**) Significance analysis of fluorescence intensity at varying concentrations of MPXV. (**c**) Standard curve of monkeypox fluorescence intensity.

In 2023, the research group conducted a study on single-line competitive FLFA for detecting the monkeypox virus (MPXV) [25]. To evaluate the quantitative analysis capability of the proposed method for sandwich FLFA, we analyzed the experimental data collected in this study. Figure 7 illustrates the application of our method to this data. Figure 7a displays nine single-channel FLFA test strips, each demonstrating different fluorescence intensities. As shown in Figure 7b, the experimental data are expressed as the mean ± SEM of the results from three independent measurements. MPXV samples at different concentrations exhibited significant differences in fluorescence intensity. In the figure, * indicates a difference (*p* < 0.05) and ** indicates a significant difference (*p* < 0.01).

In the direct detection mode, as the concentration of the monkeypox virus standard decreased, the red fluorescence on the T-line gradually diminished until it was no longer visible to the naked eye (with a detection limit of approximately 0.01 ng/mL). This paper utilizes a filterless detection system and the proposed CWT analysis algorithm to enhance the sensitivity of target concentration detection. The calibration curve of the measured fluorescence signal was calculated, and the monkeypox virus samples were quantitatively analyzed on the FLFA test paper. The calibration curve, derived from the four-parameter logistic equation, is presented in Figure 7c. Based on these results, the developed detection system was employed for MPXV in this study, demonstrating effective detection within a concentration range of 0.005 ng/mL to 100 ng/mL and a sensitivity of 0.005 ng/mL (The detection limit was calculated in Supplementary Material S14). This standard curve was utilized for the quantitative analysis of monkeypox virus detection using a single-color FLFA system.

#### 3.4.2. Quantitative Analysis of Dual-Channel FLFA

(1) Sandwich FLFA

**Figure 8:**
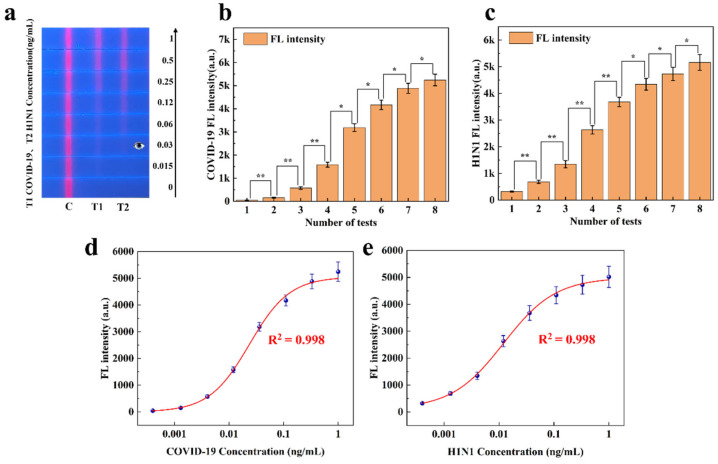
(**a**) COVID-19 and H1N1 detection strips with different fluorescence intensities. (**b**) Significance analysis of fluorescence intensity at varying concentrations of the T1 line. (**c**) Significance analysis of fluorescence intensity at varying concentrations of the T2 line. (**d**) Standard curve of COVID-19 fluorescence intensity. (**e**) Standard curve of H1N1 fluorescence intensity.

The research group conducted a double-line sandwich FLFA study for COVID-19 and H1N1 in 2023 (Figure S1). To assess the quantitative analysis capability of the proposed method for sandwich FLFA, the experimental data collected were analyzed. Figure 8 illustrates the results of applying this method to the experimental data. Figure 8a displays eight two-channel FLFA test strips, each showing different fluorescence intensities. As shown in Figure 8b,c, the experimental data are expressed as the mean ± SEM of the results from three independent measurements. COVID-19 and H1N1 samples at different concentrations exhibited significant differences in fluorescence intensity. In the figure, * indicates a difference (*p* < 0.05) and ** indicates a significant difference (*p* < 0.01).

In direct detection mode, as the concentrations of COVID-19 and H1N1 virus standards decreased, the red fluorescence on the T-line gradually diminished until it was no longer visible to the naked eye (with a detection limit of approximately 0.03 ng/mL). This paper utilizes a filterless detection system and the proposed CWT analysis algorithm to enhance the sensitivity of target concentration detection. The calibration curve of the measured fluorescence signal was calculated, and the two samples of COVID-19 and H1N1 were quantitatively analyzed on the FLFA test paper. The calibration curve, derived from the four-parameter logistic equation, is presented in Figure 8d,e. Based on these results, the developed detection system was employed for COVID-19 and H1N1 in this study, demonstrating effective detection within a concentration range of 0.015 ng/mL to 1 ng/mL and a sensitivity of 0.015 ng/mL. These standard curves enable the quantitative analysis of COVID-19 and H1N1 virus using a single-color FLFA system. By comparing the SNR of the T-line on test strips at different reaction times, we ultimately determined the optimal reaction time to be 10 min (Figure S4).

(2) Competitive FLFA

**Figure 9:**
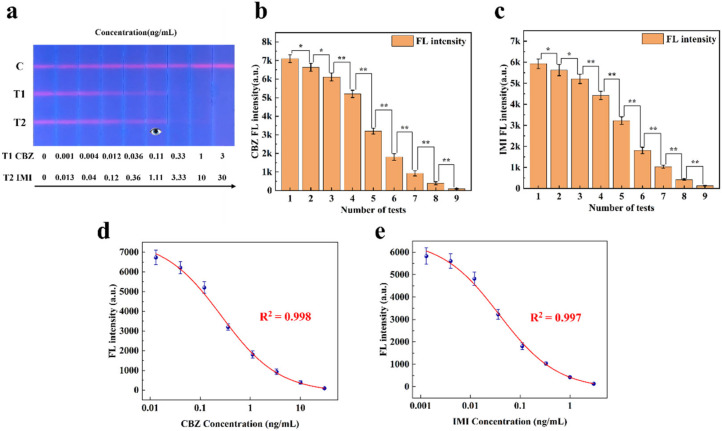
(**a**) CBZ and IMI detection strips with different fluorescence intensities. (**b**) Significance analysis of fluorescence intensity at varying concentrations of the T1 line. (**c**) Significance analysis of fluorescence intensity at varying concentrations of the T2 line. (**d**) Standard curve of CBZ fluorescence intensity. (**e**) Standard curve of IMI fluorescence intensity.

Unlike sandwich FLFA, the T-line signal intensity in competitive FLFA exhibits a negative correlation with sample concentration; in other words, higher sample concentrations lead to weaker T-line signals (for details, see Supplementary Information S6). The research group conducted a double-line competitive FLFA study for small molecules, carbendazim (CBZ) and imidacloprid (IMI), in 2023 [26]. To verify the quantitative analysis capability of the proposed method for competitive FLFA, the experimental data collected were analyzed. Figure 9 shows the results of applying this method to the experimental data. Figure 9a displays nine two-channel FLFA test strips with varying fluorescence intensities. As shown in Figure 9b,c, the experimental data are expressed as the mean ± SEM of the results from three independent measurements. CBZ and IMI samples at different concentrations exhibited significant differences in fluorescence intensity. In the figure, * indicates a difference (*p* < 0.05) and ** indicates a significant difference (*p* < 0.01).

This paper utilizes a filterless detection system and the proposed CWT analysis algorithm to enhance the sensitivity of target concentration detection. The calibration curve of the measured fluorescence signal was calculated, and the samples of carbendazim and imidacloprid were quantitatively analyzed on the FLFA test paper. The calibration curve, derived from the four-parameter logistic equation, is presented in Figure 9d,e. Based on these results, the developed detection system was utilized for the detection of CBZ and IMI in this study. CBZ exhibited a linear detection range of 0.001 ng/mL to 3 ng/mL with a sensitivity of 0.001 ng/mL. Similarly, IMI demonstrated a linear detection range of 0.013 ng/mL to 30 ng/mL, with a sensitivity of 0.013 ng/mL.

## 4. Conclusions

In this study, a dual-mode FLFA test strip signal reader was designed, which includes an image acquisition device, an image processing algorithm, a portable power conversion circuit, and dual-mode fluorescence detection software.

The multi-mode FLFA reader designed in this study can realize the quantitative detection of fluorescent substances on the reagent strip under the detection of fluorescent substance solution concentration, and the measurement results are reliable and accurate. Through experiments, we reached the following conclusions: the fluorescence detection limit of the detector can reach 10^−10^ mol/L. The standard deviation of the repetitive test of relative fluorescence intensity was lower than 0.019, and the Pearson correlation coefficient of the accuracy test was 0.9865 using the commercial FLFA reader. The correlation coefficient of the quantitative fitting curve of the actual sample was R^2^ > 0.997. The above data show that the instrument has the advantages of portability, good stability, and high precision.

Finally, built on an ARM Cortex-A76 architecture, the embedded platform implements an optimized continuous wavelet transform (CWT) algorithm while keeping hardware costs below $300 and sustaining 95% detection accuracy. Its handheld form supports one-click operation—from image acquisition to detection and reporting—in under 5 s and requires no specialized training. Consequently, the system offers a practical solution for primary healthcare settings and at-home testing. (The performance comparison of the fluorescence detection system is shown in Supplementary Material S15.)

## Figures and Tables

**Table 1:** Comparison of the accuracy of peak localization algorithms.

Method	Test Accuracy	
	Weak Signal	Noise
Threshold method	71%	65%
Curve-fitting	80%	76%
Dynamic programming	85%	80%
Gaussian model peak localization	89%	81%
Our method	95%	98%

## Data Availability

Data is contained within the article.

## Supplementary Materials

The following supporting information can be downloaded at: https://www.mdpi.com/article/10.3390/s25133846/s1. Additional experimental descriptions (S1. Materials section; S2. Preparation of experimental materials; S3. Sensitivity test of fluorescence-reading equipment; S4. Accuracy test of fluorescence-reading equipment; S5. Testing time optimization experiment; S6. Principle of the FLFA sandwich method and the competition method; S7. Experiment on signal intensity gain with and without optical filters; S8. Comparison of filtering performance among different wavelet functions in continuous wavelet transform; S9. Comparison of peak localization ability between CWT and cumulative derivation algorithm; S10. Selection of thresholds for effective peaks; S11. Scaling analysis of wavelet basis functions; S12. Excitation spot coverage design and light intensity stability calibration; S13. Excitation-emission spectral characteristics of BP365-40K filters and quantum dots; S14. Concentration setting standard and LOD calculation method; S15. Quantification of the noise suppression effect of CWT; S16. Performance evaluation of a portable fluorescence detection system. The supporting Figures and Tables referenced in the main text include Figures S1–S8 and Tables S1–S8) (References [25,26,27,28,29,30,31,32,33] are cited in the Supplementary Materials).
