# Supplementary material for: Continuous Wavelet Transform-Based Method for High-Sensitivity Detection of Image Signals of Fluorescence Lateral Flow Assay
Source: Sensors (Basel). 2025 Jun 20;25(13):3846. doi: 10.3390/s25133846 (PMC12252362; doi:10.3390/s25133846)
Supplement: Supplementary file 1 [file sensors-25-03846-s001.zip › sensors-3618168-supplementary.pdf]

Supplementary Material

Continuous Wavelet Transform-Based Method for High Sensitivity Detection of Image Signals of Fluorescence Lateral Flow Assay

Tao Zhang[1], Xiaosong Wu[2], Qian Wang[2], Long Zhang[2], Zhigang Li[2], Yangyang Peng[2], Qian Bian[1], Hui Shi[1], Yong Liu[1,2,*], Shu Wang[2,*]

[1] School of Biomedical Engineering, Anhui Medical University, Hefei 230032, PR China

[2] Hefei Institute of Physical Science, Chinese Academy of Sciences, Hefei 230036, PR China

* Corresponding author

Email: liuyong@aiofm.ac.cn (Y.L.);

Email: wangshu@aiofm.ac.cn (S.W.)

Supplementary information contents

S1. Materials section

S2. Preparation of experimental materials

S3. Sensitivity test of fluorescence-reading equipment

S4. Accuracy test of fluorescence-reading equipment

S5 Testing time optimization experiment

S6. Principle of the FLFA sandwich method and the competition method

S7. Experiment on signal intensity gain with and without optical filters

S8. Comparison of filtering performance among different wavelet functions in continuous wavelet transform

S9. Comparison of peak localization ability between CWT and the cumulative derivation algorithm

S10. Selection of thresholds for effective peaks

S11. Scaling analysis of wavelet basis functions

S12. Excitation spot coverage design and light intensity stability calibration

S13. Excitation-emission spectral characteristics of BP365-40K filters and quantum dots

S14. Concentration setting standard and LOD calculation method

S15. Quantification of the noise suppression effect of CWT

S16. Performance evaluation of a portable fluorescence detection system

Supplement **Figure S1-S8 Table S1-S8**

S1. Materials section

S1.1 Chemicals and reagents

Carboxyl-functionalized red CdSe/ZnS-625-MPA quantum dots (R-QDs) were purchased from Meiso Optoelectronics Co., Ltd. (Suzhou, China). Ammonia and anhydrous ethanol were supplied by Aladdin Biochemical Technology Co., Ltd. (Shanghai, China) and Shanghai Titan Technology Co., Ltd. Four ethoxy silane (TEOS), branched polyethyleneimine (PEI), 2-(N-morpholino) ethanesulfonic acid (MES), phosphate-buffered saline (PBS), Tween-20, N-(3-dimethylaminopropyl)-N'-ethylcarbodiimide hydrochloride (EDC), N-hydroxysuccinimide (NHS), bovine serum albumin (BSA), and fetal bovine serum (FBS) were all sourced from standard suppliers. Methyl thiophanate (CBZ) and imidacloprid (IMI) were purchased from Macklin Inc. (Shanghai, China). Anti-KAN monoclonal antibodies and encapsulated antigen-KAN-BSA were provided by Qiyi Biotechnology Co., Ltd. (Shanghai, China). Anti-CBZ monoclonal antibodies and encapsulated antigen-CBZ-BSA were obtained from Acthteam (USA).

S1.2 Fluorescence reading equipment components

The SVC LED chip (365 nm UV LED) was purchased from Viosys (Seoul, Korea). The ICX285 charge-coupled device camera (CCD-CAM), 5V lithium battery, and 5-inch touch screen were obtained from Panasonic Corporation (Japan). An 8 GB Raspberry Pi 5 was acquired from the official Raspberry Pi website. The plastic casing of the device was designed and optimized using SolidWorks software, and the physical casing was produced using 3D printing technology.

S2. Preparation of experimental materials

S2.1 Synthesis of SiO_2 double quantum dot nanospheres (R-SDQD)

S2.1.1 SiO_2 nanoparticles

SiO_2 nanoparticles (~200 nm) are prepared using a seed growth strategy. A mixture of 8 mL ammonia water and 12 mL deionized water is added to 200 mL of anhydrous ethanol, followed by 8 mL of TEOS. The mixture is stirred for two hours, centrifuged to obtain the SiO_2 precipitate, washed with ethanol, and dispersed for later use.

S2.1.2 SiO_2 double quantum dot nanospheres (R-SDQD)

R-SDQD are synthesized using PEI-mediated layer-by-layer self-assembly. A 0.5 mL SiO_2 solution is added to 40 mL PEI solution, treated ultrasonically for 30 minutes, and centrifuged to obtain positively charged SiO_2-PEI spheres. R-QD is added, followed by ultrasonic treatment. The process is repeated to wrap a second layer of R-QDs, and the final R-SDQD is collected and dispersed in ethanol (Figure S1a).

S2.2 Development of FLFA test strips

S2.2.1 Preparation of test strips for COVID-19 and Influenza A

The test strip includes a sample pad, NC membrane, absorbent pad, and PVC backing. Three test lines (T1 for Influenza A, T2 for COVID-19) and one control line

(C-line) are set on the NC membrane. After preparing and spraying the antibodies, the membrane is dried, assembled, cut into 3 mm strips, and stored with a desiccant (Figure S1b).

S2.2.2 Preparation of IMI and CBZ test strips

This dual-channel test strip can detect two target pesticides. Two test lines (T1 for IMI-BSA and T2 for CBZ-BSA) and a control line are set on the NC membrane. The antibodies are sprayed, and the membrane is dried, assembled, cut, and stored similarly to the COVID-19 and Influenza A test strips.

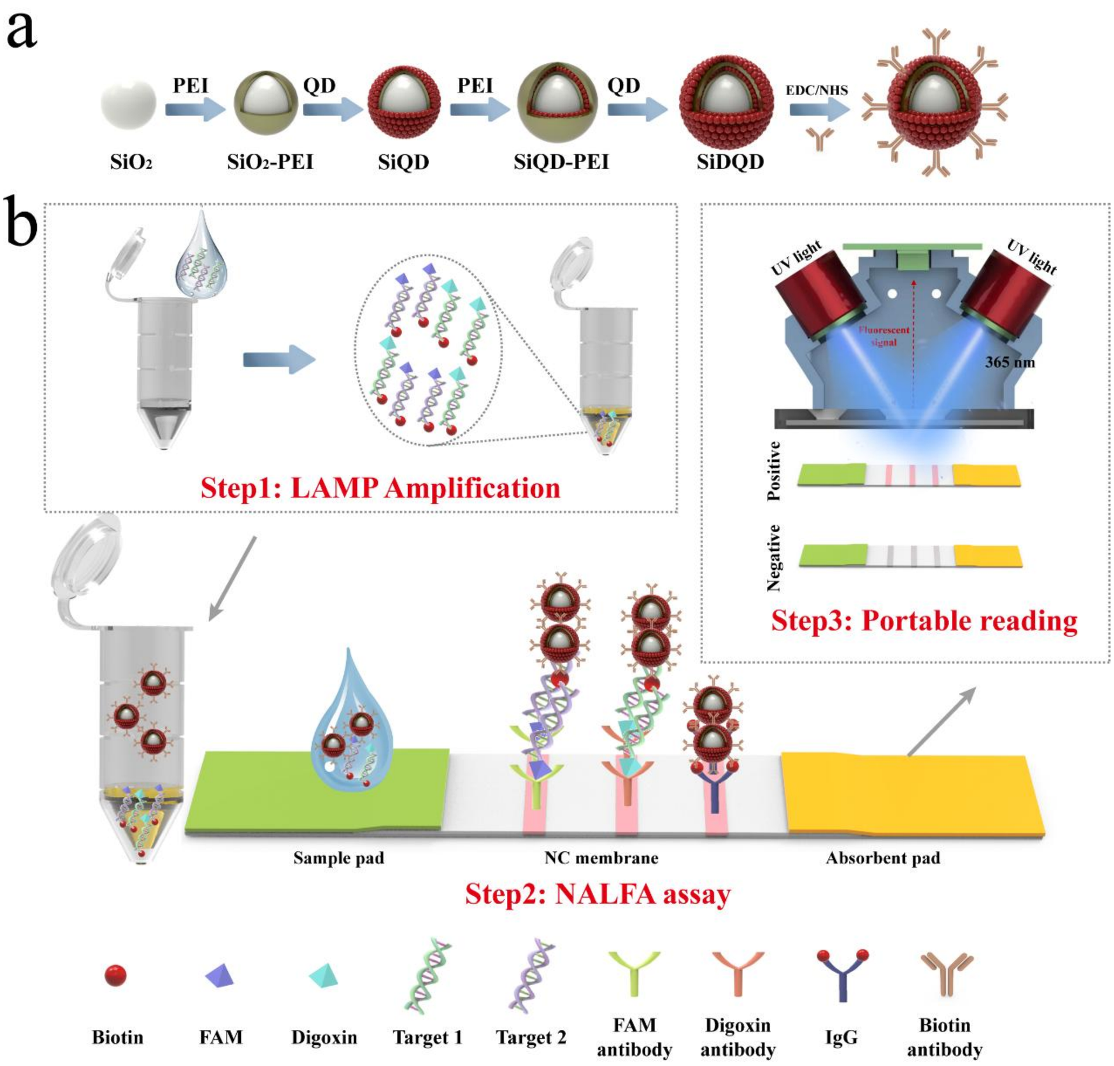

Figure S1. Schematic diagram of the FLFA principle. (a) Preparation of SDQD. (b)

Preparation of FLFA test strips.

S3. Sensitivity test of fluorescence-reading equipment

Since this system does not include a filter, the camera spectrum acquisition range extends from 420 nm to 750 nm. As shown in Figure S2, in the fluorescence detection capability of the system, we employed a fluorescence dye method to determine the minimum detection sensitivity of the optical system. Red CdSe/ZnS quantum dot solutions, used for labeling, were tested. The excitation wavelength was 365 nm, and the emission wavelength was 625 nm. Quantum dot solutions were prepared at concentrations ranging from 10^{-10} to 10^{-2} mol/L. Nine groups of fluorescent bands with signal gradients were prepared on a nitrocellulose membrane using the QG001 high-speed continuous strip cutter and gold jet scribing instrument. The signals were read using our designed algorithm under 365 nm laser excitation. S-type curve-fitting analysis revealed a significant correlation between quantum dot signals across concentrations ranging from $10^{-10}$to 10^{-2} mol/L. The fitting curve for the relationship between quantum dot solution concentration and fluorescence value was $y = 5039.37 \pm -5157.87 / [1 + (x/7.12E\text{-}7)^{0.39}]$, with a correlation coefficient of $R^2 = 0.994$ (Figure S2). The detection limit (LOD) of the target, calculated using the function, was determined to be 10^{-10}mol/L. The algorithm significantly enhances the signal-to-noise ratio of the fluorescence signal, effectively improving the detection limit.

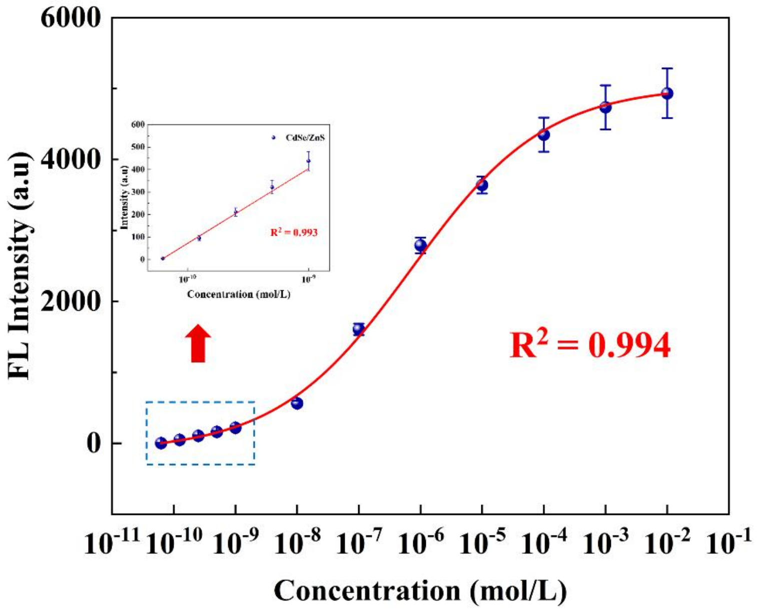

Figure S2. Fluorescence quantitative reader fluorescence intensity detection sensitivity test.

S4. Accuracy test of fluorescence-reading equipment

To evaluate the fluorescence intensity detection accuracy of the developed fluorescence quantitative reader, we prepared eight negative sample solutions. Carbendazim antibody, modified with SDQD (Specific Dye Quantum Dot) quantum dot material (excitation wavelength: 365 nm), was used as a fluorescence probe. Each sample was analyzed using both the reader designed in this study and the commercial Helmen dry fluorescence immunoanalyzer (FIC-S1). The fluorescence intensity values measured by both instruments were recorded. As shown in Figure S3, the fluorescence intensity detection trends of the developed reader are consistent with those of the commercial instrument. After fitting the data, the Pearson correlation coefficient was calculated to be 0.9865 (with a value range from 0 to 1, where values closer to 1 indicate stronger correlation). This high correlation indicates that the performance of the fluorescence quantitative reader developed in this study is

comparable to that of the commercial instrument, demonstrating good detection accuracy.

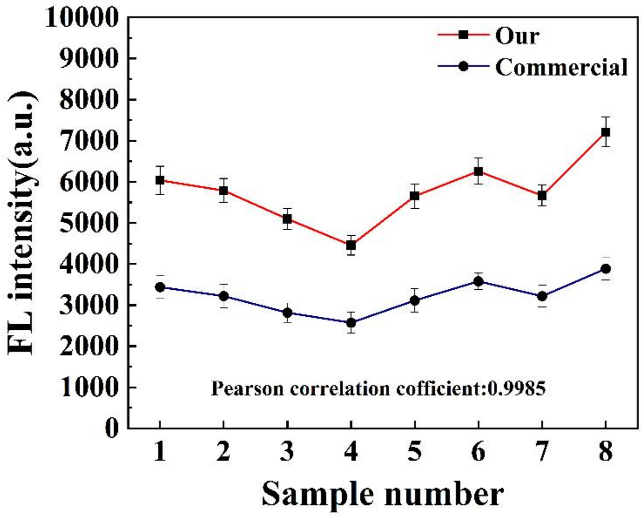

Figure S3. Accuracy test of fluorescence intensity detection by fluorescence quantitative reader.

S5. Testing time optimization experiment

To improve the speed and efficiency of detection while ensuring accuracy, we optimized the timing for fluorescent chromatographic detection of LAMP amplification products (RecA gene and blaOXA-23 gene). By comparing the SNR of the T-line on test strips at different reaction times, we ultimately determined the optimal reaction time to be 10 minutes（Figure S4）.

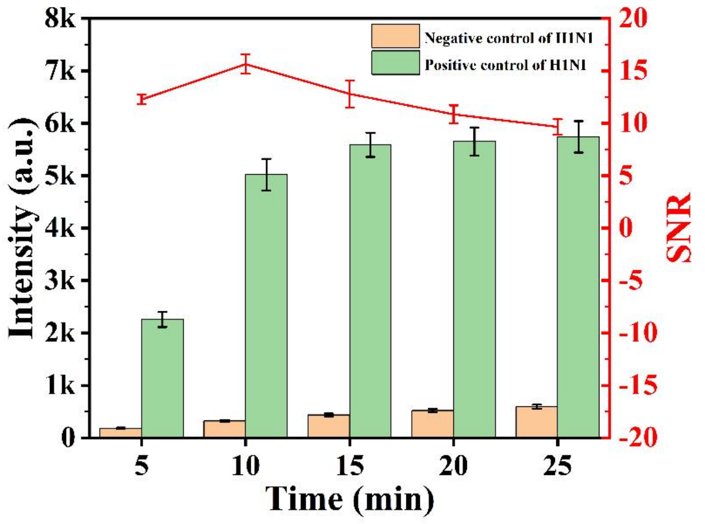

Figure S4. Reaction time optimization results.

S6. Principle of FLFA sandwich method and competition method

(1) Sandwich detection mode

This model is particularly suitable for polymer analytes with multiple test zones, such as bacteria and viruses. It employs a double antibody sandwich structure: the labeled antibody and the signal label bind to the conjugate pad, while the detection antibody is fixed on the test line (T-line), and the anti-immunoglobulin antibody is anchored at the quality control line (C-line). During the chromatography process, the target analyte forms a "labeled antibody-antigen-detection antibody" sandwich complex through bridging, resulting in a visible color change at the T-line. Excess signal labels continue to migrate and bind to the C-line. A double line color indicates a positive result, while a single color on the C-line indicates a negative result. This model is widely used for detecting pathogenic microorganisms in food [1].

(2) Competitive detection mode

Competitive mode immunochromatography is suitable for the detection of low-molecular-weight small molecule analytes, which often have only one antigenic

determinant. Since small molecules often lack the sites required for antibody binding, it is often necessary to couple them to larger proteins, such as BSA. There are two main designs of competitive analysis:

In the first design, the specific antibody is labeled on the signal tag, while the antigen–protein conjugate is immobilized on the detection line. If the target analyte is not present in the sample, two lines are displayed (negative results). When the target analyte is present, the detection line weakens or disappears. In the second design, the antigen-protein conjugate is labeled with the signal tag, while the specific antibody is immobilized on the detection line. In this case, if the target analyte is present in the sample, only the quality control line will be visible. This detection paradigm is widely used in the field of food safety to identify small-molecule hazards such as pesticides, antibiotics, and mycotoxins [2].

S7. Experiment on signal intensity gain with and without optical filters

To enhance the sensitivity of the FLFA while ensuring accuracy, a filter-free device structure design combined with the CWT algorithm was adopted. By comparing the differences in fluorescence intensity of the T-line on test strips with and without filters, it was ultimately determined that the test strip without a filter exhibited higher fluorescence intensity than the one with a filter (Table S1).

Table S1 Signal intensity gain with or without a filter.

	No filter	Add filter	Signal Strength Gain
1	794±21	55±8	739±29
2	1665±46	930±32	735±78
3	3092±131	2328±96	764±227
4	4726±165	4310±128	416±293
5	5476±213	5212±172	264±385

S8. Comparison of filtering performance among different wavelet functions in continuous wavelet transform

This study compares the effectiveness of three wavelet functions (Morlet, Gaus4, and Mexican Hat) in denoising fluorescence signals. Panel A illustrates the fluorescence signal following the addition of noise, which introduces significant noise and fluctuation. After denoising with the Morlet wavelet (Figure S5b), residual noise and multiple peaks are still evident, making it challenging to identify the effective signal. The Gaus4 wavelet (Figure S5c) reduces signal fluctuation and exhibits greater stability overall; however, invalid peaks persist, which may lead to misidentification. The Mexican Hat wavelet (Figure S5d) effectively removes redundant noise, and the detail coefficients clearly delineate the main features without invalid peaks, demonstrating superior denoising capability, particularly in the high-frequency range. A comprehensive comparison indicates that the Mexican Hat wavelet is markedly superior to the other two in denoising FLFA signals, exhibiting excellent signal clarity and denoising efficacy.

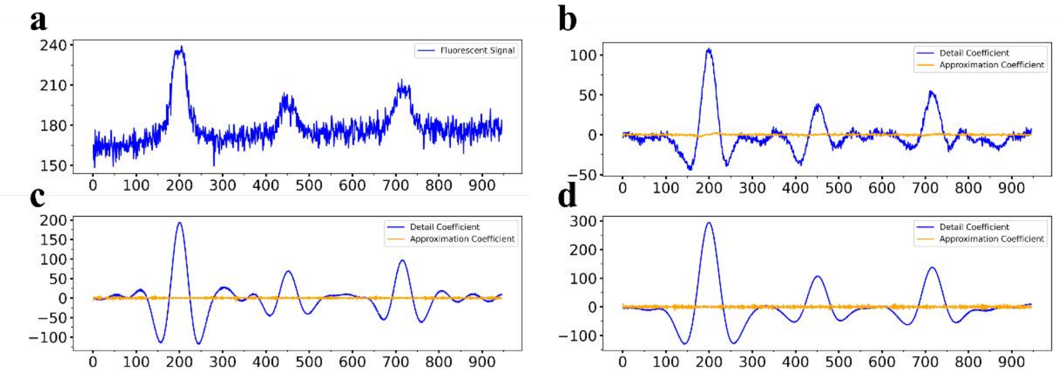

Figure S5. Denoising effects of different wavelet functions. (a) Noise-FLFA signal; (b) Morlet wavelet denoising effect; (c) Gaus4 wavelet denoising effect; (d) Mexican Hat wavelet denoising effect.

S9. Comparison of peak localization ability between CWT and the cumulative derivation algorithm

(1) Evaluation of detection algorithms for FLFA test strips

To evaluate the detection capability of the proposed algorithm, we compare its performance with the cumulative sum derivative method, using the improved wavelet transform combined with local extreme value detection. With regard to the fluorescence signals of the test strips presented in Figure S6, the details regarding sample concentration and the signal-to-noise ratio are displayed in Tables S2 and S3.

Figure S6aI and Figure S6bI illustrate the fluorescence test strips with weak signals and high-frequency noise, respectively. Figure S6aII and Figure S6bII display the wavelet coefficients of the weak fluorescence signal and the high-frequency noise signal after wavelet transformation. The wavelet transformation effectively "enhances" the curvature changes in the original signals, accentuating the low-frequency characteristics of the signals while suppressing high-frequency noise. This results in more pronounced local extreme points in the wavelet-transformed data.

Although the cumulative sum derivative method can identify some weak peak regions, it often fails to accurately detect certain peaks, as shown in Figure S6a III. Additionally, this method may detect numerous spurious peaks due to high-frequency noise interference, as depicted in Figure S6bIII. In contrast, our proposed method not only accurately detects the peaks of weak fluorescence signals, as shown in Figure S6aIV, but also effectively identifies peaks under high-frequency noise interference, as demonstrated in Figure S6bIV.

By conducting low-fluorescence detection experiments with various detection algorithms, we assessed their applicability and performance in low-light scenarios. The results indicate that the local peak detection algorithm based on wavelet transform exhibits superior sensitivity in detecting weak fluorescence signals or noise on the test strip. Consequently, this wavelet transform-based local peak detection algorithm demonstrates greater feasibility and effectiveness for detecting FLFA strips in challenging low-fluorescence conditions.

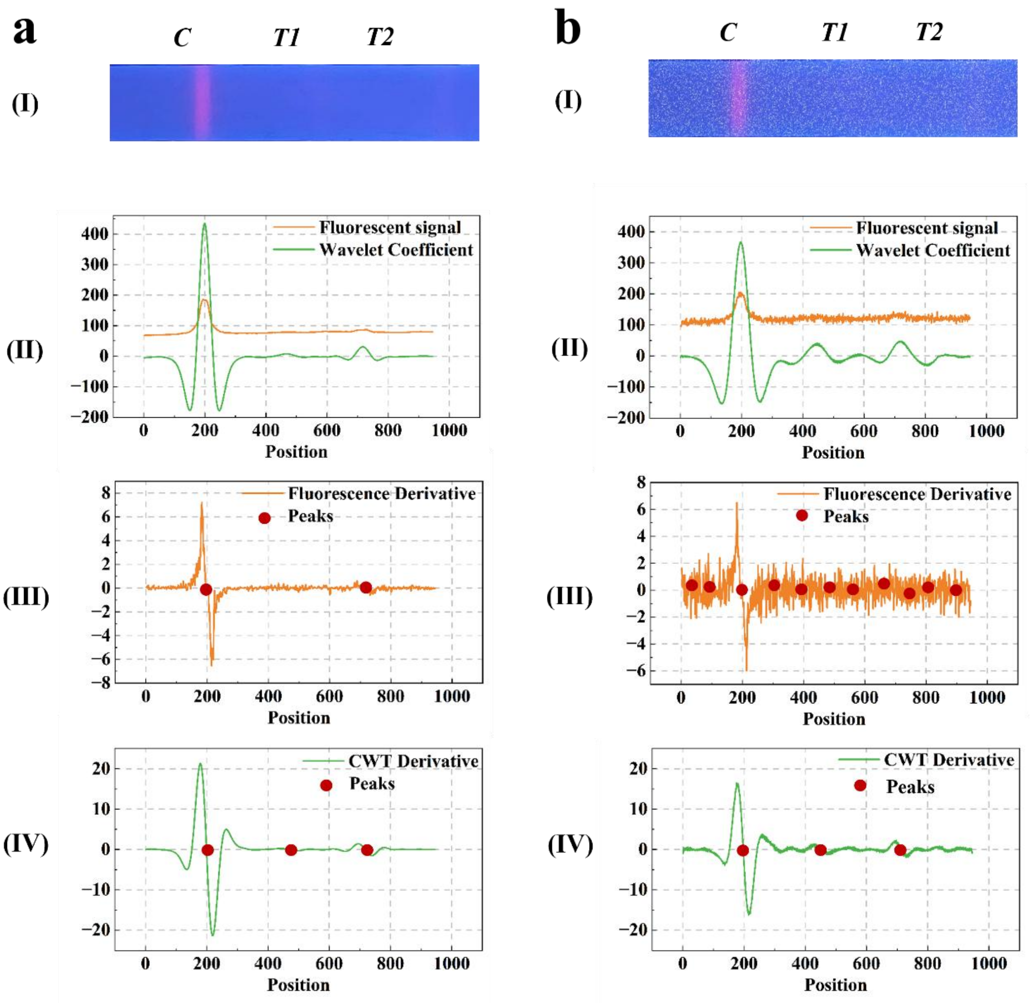

Figure S6. Comparison of peak localization ability between CWT and the cumulative derivation algorithm. (a) Comparison of peak-finding algorithms under weak fluorescence conditions. (b) Comparison of peak-finding algorithms under high-frequency noise conditions. (I) Images of test strips. (II) Projection signal and wavelet transform coefficient. (III) Peak-finding results by the cumulative derivative method. (IV) Peak-finding results of CWT.

(2) Concentration of the analyte and characterization of the signal-to-noise ratio

To evaluate the detection capability of the algorithm, the cumulative sum derivative method is employed to assess its performance against the improved wavelet

transform combined with the local extremum detection method. This study compares the capacity for detecting weak fluorescence and low signal-to-noise ratio (SNR) signals across various algorithms; monkeypox antibodies modified with silicon core double-layer quantum dot (SDQD) composite nanomaterials served as the fluorescent probe (excitation wavelength: 365 nm) for detection. Sample solutions with varying concentrations and appropriate amounts of fluorescent probes were mixed and deposited onto the test strip, and two detection algorithms were employed for analysis. The ratio of the average brightness of the test strip's T-line to the background brightness was defined as the SNR_{image} (see Tables S2 and S3).

Table S2 Weak signal test strip data parameters

	FL intensity (a. u.)	Concentration(ng/mL)	SNR_{Image}
T1	27±3	0.003	1.454
T2	116±17	0.005	1.832

Table S3 High-noise test strip data parameters

	FL intensity (a. u.)	Concentration(ng/mL)	SNR_{Image}
T1	346±21	0.01	1.121
T2	397±29	0.15	1.164

S10. Selection of thresholds for effective peaks

Data Collection: We first collected signal data from multiple experiments, focusing on the height of peak points above the baseline and the width between trough points. Systematic analysis of this data revealed the distribution characteristics of valid and invalid peaks.

Statistical Analysis: Analysis of the collected data indicated that effective peak widths range from 50 to 70, while invalid peak widths are less than 15. During data acquisition with weak signals, effective peak height and invalid peak height lack mutually applicable characteristics (Table S5).

Threshold setting: Based on the distribution of peak widths in the collected data, we established a K-nearest neighbor (KNN) classification model. A suitable value of μ, set to 32, was chosen to represent the decision boundary between effective and invalid peak widths. This method ensures that only significant peak widths are identified as valid boundaries, thereby reducing noise influence and enhancing detection reliability.

Validation: The threshold μ established using historical data was validated through multiple tests, demonstrating effective adaptability to various experimental conditions while maintaining high detection accuracy.

Table S4 Comparison of effective and invalid peak features.

Number	Effective peak height	Effective peak width	Invalid peak height	Invalid peak width
1	85.41	63	5.52	6
2	6.05	58	5.84	8
3	8.1	54	2.21	10
4	113.34	63	2.92	10
5	108.83	62	2.81	12
6	3.1	51	1.05	4
7	98.65	62	1.33	8
8	25.11	53	6.76	6
9	39.35	54	4.27	10
10	85.52	55	3.62	8
11	32.45	61	2.41	6

S11. Scaling analysis of wavelet basis functions

Through comprehensive testing and analysis of 128 scale parameters, this study demonstrates that the choice of scale interval markedly influences wavelet-transform performance (Table S5). Across all intervals examined, the 30–37-scale band exhibits a pronounced performance advantage. Although the 30–37 band comprises only eight scales (6.25 % of the total), it yields a signal-to-noise ratio (SNR) gain of 21.5–23.4 dB, a peak-position error of 0.7–1.1 pixels, and an energy concentration of 93–96 %.

Each of these three key metrics surpasses those obtained for any other interval. Specifically, relative to the adjacent 21–29 interval, the 30–37 band improves SNR gain by ≈20 %, reduces peak-position error by 40 %, and increases energy concentration by ≈8 percentage points. When the scale exceeds 50, all performance indicators deteriorate: SNR gain falls below 14.2 dB, and peak-position error rises above 2.8 pixels. These observations suggest that the 30–37 interval best matches the characteristics of typical fluorescence immunochromatography signals and, therefore, represents the optimal scale range. Moreover, the corresponding 0.25–0.32 Hz frequency band coincides precisely with the characteristic frequencies of the test strip reaction, providing a physical basis for the observed performance advantage. Accordingly, we recommend prioritizing the 30–37 scale group in practical implementations, as it maintains detection accuracy while curbing computational complexity.

Table S5. Analysis of wavelet scale performance partition characteristics

**Interval of scale**	**Number of scales**	**SNR gain (dB) range**	**Peak position error (pixels) range**	**Energy concentration (%) range**
1-20	20	5.2-12.7	3.5-8.2	45-68
21-29	9	15.3-19.8	1.2-2.1	82-89
**30-37**	**8**	**21.5-23.4**	**0.7-1.1**	**93-96**
38-50	13	18.7-20.3	1.3-1.9	85-91
51-128	78	8.5-14.2	2.8-6.5	52-75

S12. Excitation spot coverage design and light intensity stability calibration

Because the reaction zone on the test strip is 0.3 × 2 cm, whereas the excitation beam forms a 4 × 4 cm spot (Figure S7a), the beam fully envelops the target area with

ample margin. As Figure S7b illustrates, the red region in the irradiance heat map marks the illuminated zone, and the intensity across this area is uniformly distributed. To calibrate and stabilize the excitation irradiance, the UV-LED was driven by a constant-current source and mounted on a heat sink solder pad, ensuring long-term optical stability.

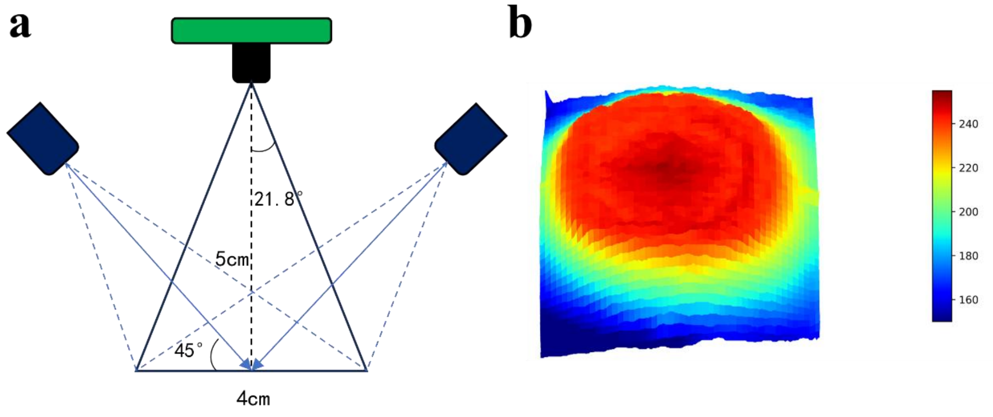

Figure S7 a. Optical path design of the detection device. b. Heat map of light intensity in the irradiation area.

S13. Excitation-emission spectral characteristics of BP365-40K filters and quantum dots

The BP365-40K narrow-bandpass filter exhibits a pronounced transmission peak at 365 nm and maintains high transmittance across the 365 ± 40 nm ultraviolet range; above 400 nm, however, transmittance declines sharply, thereby preserving the spectral purity of the excitation beam (Figure S8a; data supplied by Shijiazhuang Tangsuo Photoelectric). Absorption and emission spectra of the quantum dot solution were recorded with a microplate reader (Infinite 200 PRO M Nano). The strong absorption band of the quantum dots (300–400 nm) aligns closely with the filter's passband, whereas the characteristic emission maximum at 625 nm lies more than 200 nm away, yielding a large Stokes shift that enables efficient separation of excitation and fluorescence signals (Figure S8b).

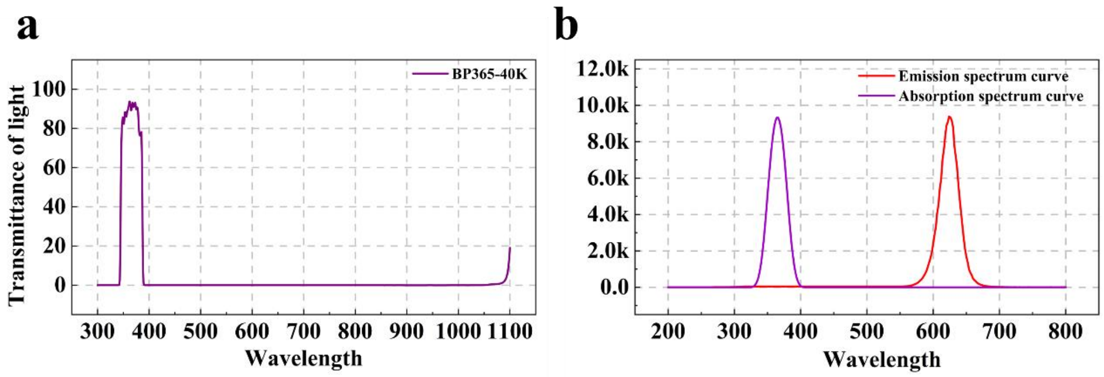

Figure S8 Spectral curves. a. Spectral curve of BP365-40K b. Emission and absorption spectral curves.

S14. Concentration setting standard and LOD calculation method

1. Principles for setting the concentration ranges of biomarkers

At the current stage, the detection ranges for the monkeypox virus (MPXV) [3], COVID-19/H1N1 [4], and agricultural residues CBZ [5] and IMI [6] are shown in Table S6. These ranges effectively cover most application scenarios, and therefore, in practical applications, we have chosen these ranges as the standard for detection.

2. Calculation Method for LOD

We follow the IUPAC-recommended "Blank + 3σ" (sandwich method) / "Blank - 3σ" (competitive method) [7,8]. The above describes the calculation methods for the LOD values of immunochromatographic signal detection using the sandwich and competitive methods.

Table S6 Biomarker concentration intervals

Detecting objects	Detection range (ng/mL)	References
MPXV	0.005-100	Wang C et al. Chemical Engineering Journal, 2023
COVID-19/H1N1	0.015-1	Lomae A et al. Talanta, 2024
CBZ	0.001-3	Wang Z et al. Microchimica Acta, 2023
IMI	0.013-30	Sun Y et al. Food Chemistry, 2021

S15. Quantification of the noise suppression effect of CWT

In this study, the performance of the continuous wavelet transform (CWT) algorithm was systematically assessed by quantifying its ability to suppress background noise at various excitation-light intensities. Experimental results indicate that, under weak, normal, and strong excitation, the CWT algorithm achieves noise-suppression rates of 67.3 %, 76.5 %, and 79.1 %, respectively, with corresponding signal-to-noise ratio (SNR) improvements of 9.7 dB, 14.3 dB, and 12.8 dB. These data demonstrate that the CWT algorithm effectively attenuates background noise across illumination conditions and yields the greatest SNR enhancement (14.3 dB) at nominal excitation intensity (Table S7). Although the suppression rate is highest (79.1 %) under high-intensity illumination, the SNR gain is slightly reduced because of signal-saturation effects. Collectively, the findings verify the CWT algorithm’s robust noise-reduction capability and environmental adaptability within fluorescence detection systems, thereby providing critical technical support for the development of high-sensitivity instrumentation.

Table S7. The suppression effect of background noise and CWT

Test conditions	Raw background intensity (ADU, mean ±SD)	CWT processing (ADU, mean ±SD)	Noise suppression ratio	Signal-to-noise Ratio (SNR) improvement
**under weak**	452±18	148±12	67.3%	9.7 dB
**normal**	893±207	210±35	76.5%	14.3 dB
**strong excitation**	1855±431	387±72	79.1%	12.8 dB

S16. Performance evaluation of portable fluorescence detection system

The fluorescence detection system developed in this study substantially outperforms existing commercial instruments and previously reported prototypes across multiple key performance metrics. Regarding sensitivity, the limit of detection (LOD) is 1.0×10^{-10} mol L^{-1}, representing an improvement of two orders of magnitude over the commercial FIC-S1 reader and one order of magnitude over the most recent report [9]. Moreover, the system exhibits a dynamic range of up to 10^6, surpassing the 10^4 range of the commercial instrument and the 10^5 range reported in the literature, thereby delivering superior linear quantitation.

With respect to throughput, the assay is completed in only 3 s, twice as fast as the commercial comparator. Although the signal-to-noise ratio (SNR) of 26 dB is marginally lower than that of single-filter commercial devices (32 dB) and recent studies (35 dB), the filter-free design still affords acceptable SNR performance (Table S8).

From a practical and economic perspective, the total hardware cost remains below USD 300—approximately one-fifth that of the commercial instrument—while retaining a portable 1 kg form factor. The platform integrates an innovative continuous wavelet transform (CWT) algorithm with a peak-finding routine. This

configuration provides greater analytical stability in complex environments than either the Gaussian-fitting approach used by commercial devices or the derivative-based peak-finding adopted in recent studies. Collectively, these advantages impart substantial application value and market competitiveness to the system in point-of-care testing (POCT).

Table S8. Comparison between existing business models and this system

parameters	Our system	Commercial equipment (FIC-S1 Reader)	Similar research
**LOD**	10^{-10}mol/L	10^{-8}mol/L	10^{-9}mol/L
**Dynamic range**	10^{6}	10^{4}	10^{5}
**Signal-to-noise Ratio**	26 dB (No filter)	32 dB (Add filter)	35 dB (Add filter)
**Detection time**	3 s	6 s	5 s
**Cost (USD)**	<300	1,500	1,200 (Prototype machine)
**Portability**	Hand-held (1 kg)	portable (3 kg)	Hand-held (1.5 kg)
**Algorithm core**	CWT+ Peak-finding algorithm	Gaussian fitting	Take the derivative and find the peak

References

1. Hsieh, H. V.; Dantzler, J. L.; Weigl, B. H. J. D., Analytical tools to improve optimization procedures for lateral flow assays. **2017,** *7* (2), 29.
2. Wang, S.; Liu, Y.; Jiao, S.; Zhao, Y.; Guo, Y.; Wang, M.; Zhu, G. J. J. o. a.; chemistry, f., Quantum-dot-based lateral flow immunoassay for detection of neonicotinoid residues in tea leaves. **2017,** *65* (46), 10107-10114.
3. Wang C, Yu Q, Li J, et al. Colorimetric–fluorescent dual-signal enhancement immunochromatographic assay based on molybdenum disulfide-supported quantum dot nanosheets for the point-of-care testing of monkeypox virus. **2023**, 472: 144889.
4. Lomae A, Teekayupak K, Preechakasedkit P, et al. Peptide nucleic acid probe-assisted paper-based electrochemical biosensor for multiplexed detection of respiratory viruses. **2024**, 279: 126613.
5. Wang Z, Zheng S, Zhang C, et al. Introduction of multilayered quantum dot nanobeads into competitive lateral flow assays for ultrasensitive and quantitative monitoring of pesticides in complex samples. **2023**, 190(9): 361.
6. Sun Y, Zhang N, Han C, et al. Competitive immunosensor for sensitive and optical anti-interference detection of imidacloprid by surface-enhanced Raman scattering **2021**, 358: 129898.
7. Wang C, Shen W, Li Z, et al. 3D film-like nanozyme with a synergistic amplification effect for the ultrasensitive immunochromatographic detection of respiratory viruses. **2024**, 18(37): 25865-25879.
8. Bai W, Zheng S, Li Z, et al. Silicon-based bimetallic nanozyme-enhanced immunochromatographic strips for highly sensitive simultaneous detection of multiple environmental pollutants. **2025**: 159936.
9. Wang Z, Zheng S, Wang C, et al. A novel competitive color-tone change fluorescence immunochromatographic assay for the ultrasensitive detection of pesticide and veterinary drug residues. **2024**, 417: 136125.
